# Prenatal Diagnosis of a Segmental Small Bowel Volvulus with Threatened Premature Labor

**DOI:** 10.1155/2017/7642784

**Published:** 2017-11-02

**Authors:** Barbara Monard, Nicolas Mottet, Rajeev Ramanah, Didier Riethmuller

**Affiliations:** ^1^Obstetrics and Gynecology Department, Besancon University Medical Center, 3 boulevard Alexandre Fleming, 25000 Besancon, France; ^2^University of Franche-Comte, Hauts de Chazal, 19 rue Ambroise Paré, 25000 Besancon, France

## Abstract

Fetal primary small bowel volvulus is extremely rare but represents a serious life-threatening condition needing emergency neonatal surgical management to avoid severe digestive consequences. We report a case of primary small bowel volvulus with meconium peritonitis prenatally diagnosed at 27 weeks and 4 days of gestation during threatened premature labor with reduced fetal movements. Ultrasound showed a small bowel mildly dilated with thickened and hyperechogenic intestinal wall, with a typical whirlpool configuration. Normal fetal development allowed continuation of pregnancy with ultrasound follow-up. Induction of labor was decided at 37 weeks and 2 days of gestation because of a significant aggravation of intestinal dilatation appearing more extensive with peritoneal calcifications leading to the suspicion of meconium peritonitis, associated with reduced fetal movements and reduced fetal heart rate variability, for neonatal surgical management with a good outcome.

## 1. Introduction

Intestinal volvulus is a serious life-threatening disease and it can cause severe digestive consequences in children. Prenatal diagnosis is very difficult because its occurrence is rare and its nonspecific clinical signs are variable. Intestinal volvulus is commonly associated with malrotation or atresia. A primary small bowel volvulus is extremely rare. Prenatal diagnosis enables appropriate surgical management immediately after birth and improves neonatal outcome. We report a case of prenatally diagnosed primary small bowel volvulus with meconium peritonitis at 27 weeks and 4 days of gestation during threatened premature labor with a good outcome.

## 2. Case Report

**Figure 1:**
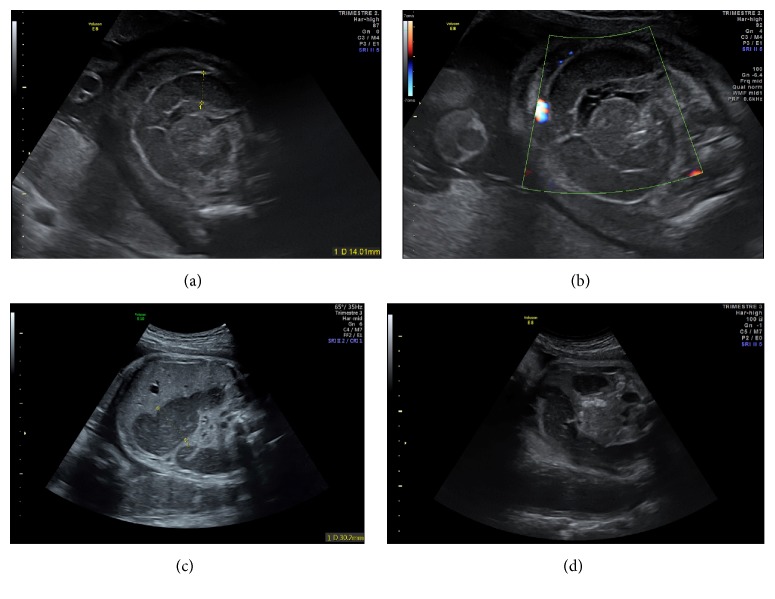
Mildly dilated small bowel with thickened and hyperechogenic intestinal wall (a) in a typical whirlpool configuration (a, b). Aggravation of intestinal dilatation (c) and peritoneal calcifications leading to the suspicion of meconium peritonitis (d).

**Figure 2:**
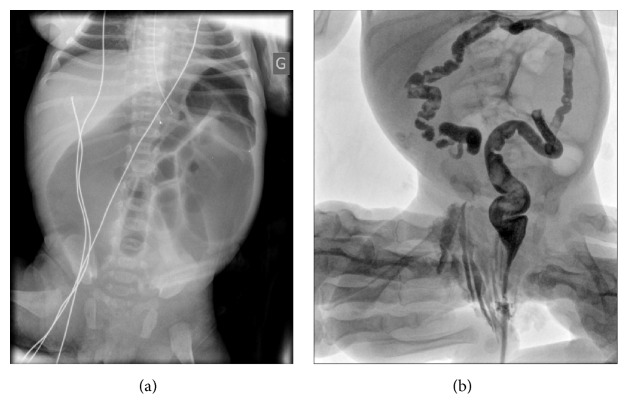
Postnatal plain abdominal X-ray showing a voluminous dilated bowel loop (a). Water-soluble contrast enema with opacification of a few centimeters of the last ileal loop (b).

**Figure 3:**
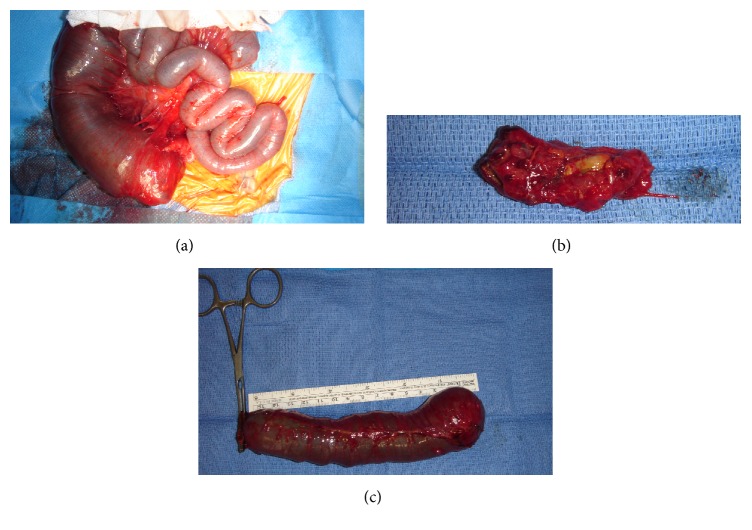
Small bowel atresia with dilated small bowel five centimeters above ileocaecal valve (a). Volvulus loop resected (b) and 16 cm of dilated small bowel resected (c).

A 30-year-old primiparous woman was seen at our hospital for preterm contractions for seven days with reduced fetal movements at 27 weeks and 4 days of gestation. A threatened premature labor was diagnosed. Ultrasound showed a female fetus with normal development and amniotic fluid volume. Small bowel appeared mildly dilated (14 mm) with thickened and hyperechogenic intestinal wall. There was a typical whirlpool configuration of the bowel (Figure 1). First and second trimester ultrasounds were unremarkable. Screening for infectious diseases was negative. A molecular genetic testing of* CFTR* was realized in the parents who were tested for the 32 main mutations of* CFTR* during a genetic counseling. This testing was negative in both of the parents. So, the fetus was not screened for cystic fibrosis. The patient received atosiban for tocolysis and steroids for fetal lung maturation. Ultrasound follow-up one week later and every two weeks showed absence of significant modification in small bowel dilatation and normal fetal development and amniotic fluid volume up to 33 weeks and 1 day of gestation when a peritoneal calcification appeared leading to the suspicion of meconium peritonitis. Fetal biometry measures including the abdominal circumference and amniotic fluid volume were normal throughout the follow-up in antepartum period. Ultrasound follow-up at 36 weeks and 4 days of gestation revealed a significant aggravation of intestinal dilatation (30 mm) appearing more extensively with persistent intestinal peristalsis, and some parietal calcifications appeared with a meconium pseudocyst but there were no ascites (Figure 1). Fetal vitality was good with a satisfying Manning's score; there were neither ascites nor significant increasing in abdominal circumference nor abnormality in fetal heart rate and the amniotic fluid volume was normal. So, the patient was hospitalized for close monitoring of fetal heart rate. After consultation with members of pediatric surgery team, induction of labor was decided at 37 weeks and 2 days of gestation given the worsening ultrasound images associated with reduced fetal movements and reduced fetal heart rate variability for neonatal surgical management. Furthermore, the patient had a favorable Bishop score of 6 on clinical examination. A 2,470 g girl was born vaginally with vacuum assistance at 37 weeks and 2 days of gestation, with Apgar scores of 3, 7, and 10 at 1, 3, and 5 minutes, respectively. The neonate was ventilated for three minutes after birth with good neonatal adaptation. She received a nasogastric tube and was immediately hospitalized in pediatric intensive care unit. Her vital and biological parameters were normal except for hemoglobin. The newborn was mildly anemic with a hemoglobin level of 15 g/dl. She had neither hyperthermia nor biological inflammatory syndrome (leukocyte count = 12,0 × 10^9^/l, CRP < 2,9 mg/l). Clinical examination showed no abnormality with an abdomen soft and painless on palpation but slightly distended. The postnatal plain abdominal X-ray showed a voluminous dilated bowel loop (Figure 2). The water-soluble contrast enema revealed a vacuous colon in normal position, a caecum in the right iliac fossa, and an opacification of a few centimeters of the last ileal loop (Figure 2). A right transverse laparotomy was performed the day after birth and revealed a segmental small bowel volvulus with a perforated meconium pseudocyst secondary to in utero perforation of distal ileum and a type II small bowel atresia five centimeters above ileocaecal valve (Figure 3). The residual length of small bowel was sufficient with 100 cm above atresia and 4 cm below atresia. No microbiological test has been performed during surgery because there was no sign of extensive inflammation. The meconium pseudocyst, the volvulus loop, and 16 cm of very dilated and unstressed small bowel were resected. The diameter of the loop below atresia was much smaller but ileocaecal valve was permeable. Given the significant difference in the diameter of the two loops, the distal loop was opened on its antimesenteric side to realize a termino-terminal ileoileal anastomosis more congruent without perioperative complication (Figure 3). Immediate postoperative care was simple. Recovery of bowel movements occurred two days after surgery and a normal diet with breast milk was started three days after surgery. The Guthrie (neonatal heel prick) test was negative. The anatomopathological examination revealed peritonitis signs on the serosa and the mesentery of the surgical specimens in the form of more or less voluminous calcifications. Moreover, there was panparietal ischemic necrosis of the mucosa and all other layers of the intestinal wall. Finally, there was diffuse vascular congestion and stigma of intraparietal hemorrhage. A satisfying weight curve permitted her return home thirteen days after surgery. One year after surgery, feeding and bowel movements were normal with a good growth. She did not suffer from short bowel syndrome.

## 3. Discussion

Fetal intestinal volvulus is rare and several modes of diagnosis are described in the literature. It can be diagnosed during a routine ultrasound [1] or if the patient presents clinical signs. The most common signs are reduced fetal movements and fetal heart rate abnormalities [2–7]. Another symptom frequently described in the literature is the presence of uterine contractions with or without associated threatened premature labor [8, 9]. De Felice et al. proposed an explanation of relationship between intrauterine midgut volvulus and preterm delivery. Acute fetal stress would activate both the fetal-placental adrenal and hypothalamic stress hormones, leading to premature uterine activity and preterm delivery [10]. Many of these symptoms led to prenatal diagnosis in our case report.

Prenatal ultrasound signs commonly described in the literature are polyhydramnios, hyperechogenic and dilated bowel loop, typical whirlpool sign, fetal ascites, peritoneal calcifications, and meconium peritonitis with stopped, persistent, or intensive intestinal peristalsis [1–9, 11–13]. In our case, we showed small bowel dilatation with thickened and hyperechogenic intestinal wall and there was a typical whirlpool configuration of the bowel at diagnosis. During the ultrasound follow-up, peritoneal calcifications appeared and led to suspect a meconium peritonitis. Amniotic fluid volume was normal and intestinal peristalsis was persistent.

Despite a significant aggravation of intestinal dilatation at 36 weeks and 4 days of gestation, we decided to continue pregnancy. Indeed, recurrence of intestinal dilatation if isolated was not a sufficient argument to justify premature birth. There was no other ultrasound sign of poor prognosis; fetal vitality was good with a satisfying Manning's score; there were neither ascites nor significant increasing in abdominal circumference nor abnormality in fetal heart rate. Moreover, the gestational age of birth in these children is determinant in postnatal evolution. In our team, planned vaginal delivery is considered in fetuses with gastrointestinal malformations if there is no major abnormality in fetal heart rate.

In all the cases of intestinal volvulus described in the literature, an early neonatal laparotomy was performed with intestinal resection and either termino-terminal anastomosis at the same time [1, 3, 14–17] or temporary enterostomy with secondary restoration of intestinal continuity [4, 6, 11, 18–20]. According to Raherison et al., ileostomy seems to be the best option in case of intestinal perforation or necrosis, or if there is atresia associated with peritonitis, because of a high risk of anastomotic leaks in an inflammatory or septic context. A resection with anastomosis at the same time can be performed if necrosis is restricted without peritonitis [14]. However, if there is a complete ischemic damage, simple detorsion and closing without pressure followed by a secondary reevaluation seem to be the best choice to limit intestinal resection in case of partial recovery seen during reevaluation [3, 13]. In our case, we performed an intestinal resection with termino-terminal anastomosis at the same time because there was no sign of extensive inflammation, without postoperative complication.

Intestinal volvulus and atresia often coexist [7, 14, 15]. When intestinal volvulus occurs in utero and is complicated with ischemic necrosis, secondary intestinal atresia can appear [7, 13, 14]. Primary intestinal atresia can be complicated with volvulus because of an increased peristalsis within dilated bowel close to atresia [14, 15]. According to Raherison et al., volvulus would be secondary when there is a 180- or 360-degree torsion of a dilated bowel loop above atresia, and volvulus would be primary leading to ischemia and atresia below the volvulus intestinal loop when a necrotic intestinal portion is located in the aplomb of atresia [3, 14]. This latter explanation seems to be the most probable hypothesis in our case report.

Despite a few cases of neonates with postoperative short bowel syndrome [11, 13], long-term prognosis was good overall with normal growth and feeding [1, 4, 6, 11, 13–16, 18, 20].

## 4. Conclusion

Prenatal small bowel volvulus should be suspected in case of reduced fetal movements associated with small bowel dilatation with or without polyhydramnios, especially if there are abnormalities in fetal heart rate or in a context of threatened premature labor. It is a life-threatening condition requiring an emergency neonatal surgical management with a good prognosis and a survival rate greater than 95% in case of isolated abnormality. Therefore, its early detection is important to provide for ultrasound follow-up and to reduce its morbidity and mortality. The outcome depends on the amount of residual bowel and the gestational age at the time of the event. It is a third trimester condition, when fetal lung maturity is sufficient. However, there is no prenatal imaging for assessing the risk of short bowel syndrome.
